# Socioeconomic position and childhood sedentary time: evidence from the PEACH project

**DOI:** 10.1186/1479-5868-10-105

**Published:** 2013-09-04

**Authors:** Richard M Pulsford, Pippa Griew, Angie S Page, Ashley R Cooper, Melvyn M Hillsdon

**Affiliations:** 1 Sport and Health Sciences, College of Life and Environmental Sciences, University of Exeter, Exeter, Devon, United Kingdom; 2 Centre for Exercise, Nutrition & Health Sciences, School for Policy Studies, University of Bristol, Bristol, UK

**Keywords:** Sedentary behaviour, Accelerometer, Socioeconomic position, Household income, Area deprivation, Car ownership, Parental education, Private gardens

## Abstract

**Background:**

Associations between socioeconomic position (SEP) and sedentary behaviour in children are unclear. Existing studies have used aggregate measures of weekly sedentary time that could mask important differences in the relationship between SEP and sedentary time at different times of the day or between weekdays and weekend days. These studies have also employed a variety of measures of SEP which may be differentially associated with sedentary time. This paper examines associations of multiple indicators of SEP and accelerometer-measured, temporally specific, sedentary time in school children.

**Methods:**

Between 2006 and 2007 sedentary time data (minutes spent below 100 accelerometer counts per minute) for weekdays before-school (7.00-8.59AM), during school-time (9.00AM-2.59PM) and after-school (3.00PM-11.00PM), and weekend days were recorded for 629 10–11 year old children using accelerometers. Ordinary least squares regression was used to examine associations with 5 indicators of SEP (area deprivation, annual household income, car ownership, parental education and access to a private garden). Covariates were; gender, BMI, minutes of daylight, accelerometer wear time and school travel method. Analyses were conducted in 2012.

**Results:**

Following adjustments for covariates, having a parent educated to university degree level was associated with more minutes of school (5.87 [95% CI 1.72, 10.04]) and after-school (6.04 [95% CI 0.08, 12.16]) sedentary time. Quartiles of area deprivation (most to least deprived) were positively associated with after-school (Q2: 4.30 [95% CI −6.09, 14.70]; Q3: 10.77 [95% CI 0.47, 21.06]; Q4: 12.74 [95% CI 2.65, 22.84]; *P*_*trend*_ = 0.04) and weekend (Q2: 26.34 [95% CI 10.16, 42.53]; Q3: 33.28 [95% CI 16.92, 49.65]; Q4: 29.90 [95% CI 14.20, 45.60]; *P*_*trend*_ = 0.002) sedentary time. Having a garden was associated with less sedentary time after-school (−14.39 [95% CI −25.14, -3.64]) and at weekends (−27.44 [95% CI −43.11, -11.78]).

**Conclusions:**

Associations between SEP and children’s sedentary-time varied by SEP indicator and time of day. This highlights the importance of measuring multiple indicators of SEP and examining context specific sedentary time in children in order to fully understand how SEP influences this behaviour. Further research should combine self-report and objective data to examine associations with specific sedentary behaviours in the contexts within which they occur, as well as total sedentary time.

## Background

The term sedentary represents a distinct class of behaviours in which sitting predominates and little energy expenditure above resting values is required (1–1.5 metabolic equivalents [METs]) [1]. In children there is evidence of prospective associations between both specific sedentary behaviours (such as TV viewing) [2-4] and total sedentary time (objectively measured using accelerometers) [5,6] with cardiometabolic risk factors, while increasing sedentary time is believed to be an underlying factor in the development of childhood obesity [7-9]. Recent reports suggest that 10 yr old boys and girls in the UK spend 7.5 and 7.7 hrs per day respectively in sedentary behaviours [10]. As sedentary behaviour has been shown to track from childhood into adolescence [11,12] a better understanding of its correlates is important.

Socioeconomic position (SEP) is an important determinant of health as it can influence an individual’s attitudes, experiences and exposure to a range of risk factors [13]. A number of studies have observed that children with a lower SEP (as defined by family income, parental education or employment, or area deprivation) engage in higher levels of screen-based sedentary behaviour [13-17]. However, while self-reported TV viewing and screen time have often been used as a marker for a broader pattern of sedentary behaviour, agreement between self-reported and total sedentary time measured objectively may be limited [18,19] and appears to vary between population subgroups [20,21].

Studies examining associations between SEP and accelerometer-defined total sedentary time in children have reported mixed results. Positive, [22] inverse, [23] and null [10,18,23-26] associations have been observed with measures including maternal education, household income and area deprivation. Such SEP indicators measure different, often related aspects of socioeconomic stratification [27] which can act at different levels (e.g. individual, household, neighbourhood), [28,29] at different time points, [30,31] and through different causal pathways [32-34]. As the choice of SEP indicator may determine the strength and direction of associations between sedentary time and health, wherever possible it is beneficial to utilise multiple indicators at more than one level [35].

Existing studies also average sedentary time across all measurement days to achieve a single measure in minutes per day and as a consequence may fail to account for variations in the association between SEP and sedentary time between weekdays and weekends and different times of the day. When considering strategies to reduce sedentary time it is important not only to know who may be most at risk but also at what period of the day these strategies might be most beneficial [36].

In order to gain a clearer understanding of the association between SEP and childhood sedentary time this study examined the influence of a range of indicators of SEP on objectively assessed sedentary time on weekdays before, during and after school, and on weekend days.

## Methods

### Participants

Thirteen hundred and seven primary school children (aged 10–11 yrs) were recruited from a large UK city as part of the PEACH project (Personal and Environmental Associations with Children’s Health). The children attended one of 23 state funded primary schools that had the highest (>40%) transition rate to 8 state funded secondary schools. These secondary schools were chosen to be representative of the city on the basis of geographic location and area deprivation. Only one primary school declined to take part in the study. The project aimed to examine the environmental and personal determinants of physical activity in children as they transition from primary schools to secondary school. Of 1899 children who were invited to take part 1340 (70.5%) agreed to take part and 1307 were present at school on the first day of measurement [37]. Informed parental consent was obtained for all children and ethical approval for the study was provided by a University of Bristol ethics committee. Baseline data collection was carried out between 2006 and 2007. Data was collected from the children during school visits and from parents using questionnaires completed at home. Further details of participants and procedures are available elsewhere [38].

### Measures

### Dependent variables

Sedentary time was assessed by waist-worn Actigraph GT1M accelerometers (Actigraph, Pensacola, Florida). This monitor has been validated [39,40] and used extensively as an objective measure of physical activity and sedentary time in children [9,41]. Children were required to wear their accelerometer during waking hours for 7 consecutive days (excluding water-based activities) during which time activity data was recorded at 10 second epochs. Data reduction was carried out using Kinesoft software. Periods of 60 minutes or more at zero accelerometer counts per minute (cpm) were considered to be non-wear time and were excluded. Sedentary time was defined as <100 cpm [42]. Total sedentary time in minutes was recorded separately for three weekday periods (before-school, 7.00AM to 8.59AM; school-time, 9.00AM to 2.59M; after-school, 3.00PM to 11.00PM) and for weekend days. A valid measurement day consisted of ≥ 600 mins of accelerometer wear time. Participants with at least one valid weekday and one valid weekend day, who had worn the accelerometer in all three weekday periods, and who had complete information on all covariates were included in the final sample (n=629).

### Explanatory variables

Parental educational attainment, annual household income, the number of cars available to the household, and whether the property has a private garden (including any outside space) were recorded by the parental questionnaire. Home postcodes for participating children were confirmed by the Local Education Authority and were used to calculate the English Index of Multiple Deprivation (IMD) score for each household. This is a neighbourhood score based on six categories of deprivation including health, income and employment [43].

Covariates included gender, accelerometer wear time (in minutes for each specific measurement period) minutes of daylight after 3.00PM, [44] method of travel both to and from school (active i.e. walk or cycle [coded 1], or non-active i.e. by car [coded 0]), and body mass index (BMI). Travel method to and from school was reported in the child questionnaire. Height and weight measures were taken on the first measurement day. Weight was measured using digital SECA scales recorded to the nearest 0.1 kilogram and height was measured using a portable Leicester height measure and recorded to the nearest millimetre. BMI was calculated as weight (kg)/height^2^(m). Analysis groups for SEP indicators and covariates are described in Table 1.

**Table 1:** Sample characteristics

**Age (yrs)**		**10.95 (0.41)**
Gender (%)	Male	50.80
	Female	49.20
Height (cm)		145.45 (7.17)
Weight (kg)		38.77 (8.36)
BMI (kg.m^2^)		18.20 (2.95)
Waist circumference (cm)		65.75 (8.03)
2007 IMD Quartile (%)	1 (Most deprived)	25.12
	2	25.44
	3	25.28
	4 (Least deprived)	24.17
Property has a private garden? (%)	No	5.66
	Yes	94.44
Annual household income (%)	<£20000	23.73
	£20-40000	39.56
	£40-60000	20.09
	>£60000	16.61
Number of cars (%)	0	7.91
	1	47.63
	2	41.61
	≥3	2.85
Parent educational level (%)	< University degree level	66.72
	≥ University degree level	33.28
Before-school		
Wear time	(mins)	68.67 (23.59)
Sedentary time	(mins)	37.0 (15.87)
School day		
Wear time	(mins)	340.91 (41.14)
Sedentary time	(mins)	218.49 (35.04)
After-school		
Wear time	(mins)	326.29 (62.83)
Sedentary time	(mins)	194.11 (47.09)
Weekend day		
Wear time	(mins)	720.69 (79.73)
Sedentary time	(mins)	440.59 (82.08)

Values are mean (SD) unless otherwise stated. N=629.

### Data handling and statistical analysis

To examine the influence of SEP on children’s sedentary time a series of ordinary least squares regression models were fitted. Each SEP indicator was entered as a categorical variable with the lowest score for each indicator (which identifies the lowest SEP) as the reference category. Robust standard errors (using the ‘cluster’ command in STATA) were applied to the analyses to take account of clustering by school.

As socioeconomic factors can interact with gender to produce different effects across groups, [45] SEP × gender interaction terms were initially added to the models. Where no significant interaction effects were apparent, gender was treated as a confounder. Initial analyses were adjusted for gender and period specific accelerometer wear time (minutes) (model 1) and then for minutes of daylight after 3pm on the first measurement day at each school [44] and BMI (model 2). As travel to and from school is a potentially important source of physical activity or sedentary time [26] analyses of before-school and after-school sedentary time was then further adjusted for school travel method to or from school respectively.

All analyses were conducted in 2012 using STATA SE version 12.1 (StataCorp, College Station, Texas) and statistical significance was set at p<0.05.

## Results

Complete information on all covariates was not available for 629 children, and a further 49 did not fulfil the wear-time criteria for these analyses. The final sample included 629 children. There were no significant differences in sedentary time before, during or after school or at weekends between those who were and those who weren’t included in the final analysis. However, those included in the analyses were more likely to be from households in less deprived areas, with a higher annual income, a greater number of cars and where a parent was qualified to university degree level or higher (*P*_*trends*_ <0.006). Accelerometer wear time was not associated with any indicator of SEP. Sample characteristics including mean accelerometer wear time and sedentary time for each measurement period are described in Table 1. Coefficients and 95% confidence intervals for models 1 and 2 are detailed in Tables 2, and 3 respectively. In order to determine how much of the association between sedentary time and SEP in the before school and afterschool periods was explained by differences in school travel method (active or non-active travel), the model 2 analysis was further adjusted for school travel. This did not significantly affect the observed associations. School travel method is therefore included as a covariate in the before-school and afterschool analysis in model 2.

**Table 2:** Sedentary time (<100 cpm) in minutes by categories of 5 indicators of SEP (model 1)

		**Sedentary behaviour (>100 counts.minute)**							
		**Before school 7–8.59AM**		**School day 9AM-2.59PM**		**After school 3-11PM**		**Weekend**	
		**Coefficient**	**95% CI**	**Coefficient**	**95% CI**	**Coefficient**	**95% CI**	**Coefficient**	**95% CI**
2007 IMD score	1 (Most deprived)	0		0		0		**0**	
(quartiles)	2	2.48	0.002, 4.97	0.96	−5.75, 7.67	4.071	−7.56, 15.70	**29.08**	**11.85, 46.30**
	3	2.08	−0.27, 4.43	0.35	−7.93, 8.62	9.65	−1.14, 20.44	**33.69**	**15.18, 52.20**
	4 (Least deprived)	1.25	−1.02, 3.52	6.79	−1.99, 15.56	12.13	1.14, 23.12	**30.22**	**12.63, 47.82**
	p_*trend*_	0.16		0.1305		0.1063		**0.0052**	
Private garden?	No	0		0		**0**		**0**	
	Yes	−1.85	−4.25, 0.55	−1.96	−9.79, 5.87	**−14.69**	**−26.04, -3.34**	**−28.19**	**−48.41, -7.97**
Number of cars	0	0		0		0		0	
	1	0.97	−1.54, 3.49	5.28	−2.95, 13.50	4.98	−3.36, 13.31	−10.57	−29.20, 8.06
	2	0.88	−2.23, 3.99	5.88	−1.62, 13.39	6.08	−3.07, 15.22	−10.66	−33.61, 12.29
	≥3	0.47	−2.61, 3.55	6.18	−8.00, 20.37	7.61	−5.63, 20.85	1.55	−24.59, 27.69
	p_*trend*_	0.7215		0.4450		0.4736		0.4987	
Household income	<£20000	0		0		0		0	
(per annum)	£20-40000	1.156	−0.26, 2.58	3.27	−1.97, 8.51	1.35	−5.31, 8.01	4.06	−16.02, 24.14
	£40-60000	−0.28	−1.92, 1.36	4.27	−3.56, 12.09	4.24	−2.45, 10.93	8.03	−13.66, 29.73
	>£60000	−1.46	−3.37, 0.46	5.58	−4.44, 15.60	2.36	−7.45, 12.16	−12.41	−30.29, 5.47
	p_*trend*_	0.10013		0.6190		0.5505		0.07	
Level of education*	< Degree level	**0**		**0**		**0**		0	
	≥ Degree level	**−1.47**	**−2.61, -0.32**	**4.60**	**0.81, 10.01**	**4.07**	**3.06, 11.21**	−0.35	−16.06, 15.35

Bold typeface indicates significance (p<0.05). Data are for participants with ≥1 valid weekday and ≥1 valid weekend day at ≥ 600 mins, and with valid wear time (>0 mins) for each weekday period. * For level of education ‘≥ Degree level’ refers to a parent being qualified to university degree level or higher. Adjusted for gender, and wear time for that period only.

**Table 3:** Sedentary time (<100 cpm) in minutes by categories of 5 indicators of SEP (model 2)

		**Sedentary behaviour (>100 counts.minute)**							
		**Before school 7–8.59AM**		**School day 9AM-2.59PM**		**After school 3-11PM**		**Weekend**	
		**Coefficient**	**95% CI**	**Coefficient**	**95% CI**	**Coefficient**	**95% CI**	**Coefficient**	**95% CI**
2007 IMD score	1 (Most deprived)	0		0		**0**		**0**	
(quartiles)	2	2.67	0.50, 4.83	0.48	−5.48, 6.45	**4.30**	**−6.09, 14.70**	**26.34**	**10.16, 42.53**
	3	2.57	0.38, 4.76	0.85	−7.20, 8.89	**10.77**	**0.47, 21.06**	**33.28**	**16.92, 49.65**
	4 (Least deprived)	1.84	−0.32, 4.00	6.14	−1.77, 14.06	**12.74**	**2.65, 22.84**	**29.90**	**14.20, 45.60**
	p_*trend*_	0.08		0.19		**0.04**		**0.002**	
Private garden?	No	0		0		**0**		**0**	
	Yes	−1.70	−3.79, 0 .40	−2.04	−8.93, 4.85	**−14.39**	**−25.14, -3.64**	**−27.44**	**−43.11, -11.78**
Number of cars	0	0		0		0		0	
	1	0.03	−2.52, 2.57	3.78	−4.65, 12.21	3.19	−4.41, 10.79	−11.33	−27.30, 4.64
	2	−0.35	−3.39, 2.69	4.38	−3.39, 12.15	4.09	−4.27, 12.46	−11.11	−31.82, 9.60
	≥3	−2.04	−4.81, 0.73	3.20	−11.15, 17.55	3.81	−8.28, 15.90	0.27	−21.59, 22.14
	p_*trend*_	0.10		0.67		0.75		0.45	
Household income	<£20000	0		0		0		0	
(per annum)	£20-40000	0.96	−0.36, 2.28	2.85	−2.19, 7.89	1.25	−5.43, 7.92	3.41	−16.24, 23.07
	£40-60000	−0.15	−1.70, 1.40	4.77	−2.80, 12.35	5.13	−1.07, 11.33	10.00	−11.16, 31.15
	>£60000	−1.21	−2.99, 0.58	6.62	−0.74, 13.99	3.99	−3.90, 11.87	−6.94	−25.24, 11.35
	p_*trend*_	0.10		0.32		0.30		0.22	
Level of education	< Degree level	0		**0**		**0**		0	
	≥ Degree level	−0.88	−2.03, 0.261	**5.87**	**1.72, 10.04**	**6.04**	**0.08, 12.16**	3.83	−13.66, 21.31

Bold typeface indicates significance (p<0.05). Data are for participants with ≥1 valid weekday and ≥1 valid weekend day at ≥ 600 mins, and with valid wear time (>0 mins) for each weekday period. * For level of education ‘≥ Degree level’ refers to a parent being qualified to university degree level or higher. All adjusted for gender, BMI, minutes of daylight after 3.00pm, and accelerometer wear time for that period. Before school and afterschool sedentary time additionally adjusted for school travel method to or from school respectively.

Having a private garden was associated with significantly lower levels of sedentary time after school and on weekend days in model 1 and remained after adjustment for all covariates (model 2). A priori, having a private garden was included as a measure of material wealth. To examine the possibility that the association between having a private garden and sedentary time is confounded by the SEP of the parents the regression models were repeated with further adjusted for household income. This made very little difference to the observed associations. Across household income groups having a private garden was still negatively associated with sedentary time. There were also no significant interactions between household income and having a private garden.

A weak inverse association was observed between parental educational attainment and sedentary time before-school and a positive association during and after school. The before-school association was attenuated to the null in model 2. The association between educational level and after-school sedentary time was modified by gender (data not shown). In girls there was no difference in recorded sedentary time by level of parental education whereas in boys higher parental education was associated with an increase in sedentary time (11.86 mins [95% CI 2.02, 21.69, *P* =0.02]).

In the fully adjusted model only, decreasing area deprivation (increasing affluence) was associated with increased after-school sedentary time. Those in the most deprived quartile also recorded a lower average sedentary time at weekends than those in less deprived groups in both model 1 and model 2.

Household income and car ownership were not associated with sedentary time during any weekday period or on weekend days across either of the 2 models and no other interaction effects were observed.

## Discussion

In order to gain a clearer understanding of the relationship between socioeconomic position and childhood sedentary time the current study sought to separately examine the influence of five indicators of SEP on objectively assessed sedentary time during three distinct periods of a school day and on a weekend day.

Having a private garden was associated with significantly less sedentary time both before and after school and at weekends. The garden is an important area for children’s outdoor play [46] and evidence suggests a negative association between outdoor play and sedentary behaviours [47,48]. It is therefore logical that having a safe private outside space would be associated with a reduction in sedentary time while children are away from school and their time is more discretionary. Although the current data refers only to private gardens it is possible that the provision of safe places to play within sight of where children live could potentially reduce sedentary time in children from households without private gardens.

During school-time children from a parent educated to university degree level or higher recorded a few more minutes of sedentary time than children from a less well educated parent. In the after-school period this relationship was only true for boys. This is consistent with one previous study which demonstrated that higher maternal education was associated with higher levels of sedentary behaviour in a large cohort of British children [22]. Method of school travel has been reported as being an important contributor to differences in sedentary time [26] in the afterschool period, but additional adjustment for active versus non-active school travel did not significantly alter the associations in the current analyses. However, it has been previously suggested that better educated parents are likely to place greater importance on their child’s academic achievements and therefore encourage more study time which could involve more sitting after school [22]. Lower SEP children may also be left unsupervised more often and for longer periods during the after-school period, possibly due to pressures of parent’s work [49]. A recent study observed that the number of hours spent unsupervised in this period was associated with higher levels of accelerometer defined physical activity, less time sitting on buses or in cars and less time sitting doing homework [49]. The gender difference in after-school sedentary time-parental education relationship is also consistent with previous evidence suggesting that boys of this age are afforded more freedom for independent mobility within the local and wider neighbourhood than girls, [38] and spend more time outdoors in the after-school period [50].

The observation that children with more educated parents record higher average school-time sedentary time is more difficult to explain although previous reports from this cohort found that children who are entitled to free school meals (an indicator of low household income/SEP) engaged in significantly greater levels of school time physical activity than those who are not [51]. Evidence also suggests that maternal education is inversely associated with disruptive behaviour [52] and symptoms of attention deficit hyperactivity disorder [53] (ADHD) in children. Although not measured in the current study it is possible that differences in school-time sitting behaviour by parental education are due to higher levels of disruptive behaviour and ADHD in children with a less well-educated parent. An alternative explanation is that children with a better educated parent attend schools that place a greater emphasis on study (sitting) time and less on play time during the school day.

During the after-school period and on weekends there was a significant increase in sedentary time in children residing in less deprived areas independent of gender, accelerometer wear time, minutes of daylight, BMI and from-school travel method (afterschool analysis only). Leventhal et al. suggest that the academic improvement observed in children of this age when they moved from areas of high deprivation to lower deprivation neighbourhoods was partially attributable to an increase in out of school study time, [54] which could provide a possible mechanism for this finding. It is also equally possible that children living in deprived neighbourhoods, in overcrowded accommodation, are permitted more independent mobility and play out more than children living in affluent neighbourhoods where parents may have greater anxiety about allowing children to play outside unsupervised.

The associations of parental education and area deprivation in the current study are not consistent with previous evidence regarding sedentary time and SEP. Specific sedentary behaviours, most frequently television or screen time, have often been associated with children in lower SEP groups [13-17] while only one other study has observed a positive association between accelerometer defined sedentary time and SEP [22]. The contrary findings in the current study may be due to methodological differences in defining the exposure (choice of SEP indicator) and the outcome (specific behaviours and total or periodic sedentary time).

A recent study reported that while TV viewing was inversely associated with indicators of SEP (including maternal education and household income) no association was evident with accelerometer defined sedentary time in children of this age group [18]. This is perhaps not surprising given that TV viewing and screen time do not adequately reflect total sedentary time in children of this age [20]. This suggests that other sitting activities (reading, homework, etc.) are important contributors to total sedentary time [21] and may be differentially associated with indicators of SEP. The differences in the results of this study highlight the specificity of the relationship between various indicators of SEP and types of sedentary behaviour and periods of time. Future research examining associations between SEP and sedentary time needs to be more specific about the hypothesised relationship between the measure of SEP employed and the type of sedentary behaviour being examined.

Accelerometer determined sedentary time is arguably more precise than self-reported sitting and allows specific time periods to be separately examined. However, methodological differences regarding the use of aggregate data, the criteria used to define sedentary time, the definition of a valid measurement day and the number of valid days analysed make comparison between studies problematic and can potentially affect the associations observed. Previous studies have used an average value for sedentary time from a minimum of three [18,23] or four [24,26] days of accelerometer data including at least one weekend day. Two of these studies defined sedentary time (<100 cpm) and a valid measurement day (≥600 minutes of valid wear time) in the same way as the current study but reported mean sedentary time values which are lower (395.5 mins [±69.3] [18] and 380 mins [±92.2] [24] compared to 449 mins [±97.96] in the current study). Both studies reported null associations between SEP and sedentary time. As a child’s choice of activity and therefore their volume of sedentary time would be more controlled during a school day than a weekend day, [10] average daily sedentary time values may be affected by the number of weekdays and weekend days from which the averages are computed and may lead to an underestimation of weekday sedentary time. The differential associations observed between sedentary time and SEP indicators in the current study, highlights the importance of examining sedentary time in different time periods separately.

This study and others demonstrate that the relationship between childhood sedentary time and SEP is a complex one. It appears that the specific sedentary behaviour being measured, the context of the behaviour (at school, at home or during different times of the day), and measure of SEP are all important factors. Future studies should combine self-report and objective measures to accurately examine specific behaviours in the context in which they occur, how they contribute to sedentary time at different times of the day and how these relationships differ across social groups using a range of relevant indicators of SEP.

### Strengths and limitations

The main limitation of this study is the potential for non-response bias due to incomplete data on indicators of SEP. However, while responders were more likely to be from a higher SEP, no significant differences in sedentary time were observed between those who were and those who were not included in the final analyses. Other limitations include the cross-sectional design and reliance on self-report data for 4 of the 5 indicators of SEP. Misclassification of the exposures (due to inaccuracies in reporting), if non-differential, would lead to an attenuation of any associations towards the null. The present study also examines sedentary time recorded between 7.00AM and 11.00PM and therefore excludes sedentary time outside of this period. This could potentially mask important associations if sedentary time before 7.00AM and after 11.00PM is socially patterned. However, in the current sample very few children recorded any data outside of this period and even fewer did so on more than one day. Defining a valid day as one with at least 600 minutes of wear time, an approach used extensively in studies examining accelerometer defined physical activity and sedentary behaviour, [18,24] also leaves the possibility that comparisons are made between individuals with data measured at slightly different times of the day.

A significant strength of this study is the large diverse sample which encompasses children from 23 different primary schools and from a range of physical and social environments. The control of known confounding factors including gender, accelerometer wear time, daylight, non-active transport and BMI is also a strength. The objective measurement of sedentary time also reduces the possibility of type two error due to misclassification associated with self-report measures. However, as discussed individual sedentary behaviours may be differentially associated with indicators of SEP, in which case a measure of total sedentary time, however accurate, may mask important associations. Despite consensus that SEP is complex and multifactorial most childrens health studies use a single socioeconomic variable to represent SEP. A key strength of the current study is the use of a broad range of robust indicators of SEP reflecting characteristics of the family, the physical household environment and surrounding neighbourhood. These indicators also have theoretically plausible pathways to the outcome.

## Conclusions

This is the first study to separately consider the influence of a range of socioeconomic factors on objectively measured sedentary time by time period. The results suggest that children of parents with a higher level of educational attainment may be more sedentary during school time and in the afterschool period in boys. Children living in less deprived areas may also be more sedentary after school and at weekends. Living in a household with a private garden was associated with a significant reduction in sedentary time outside of school hours. These differential associations between SEP and sedentary time during different periods of a school day and on weekend days are important because they highlight the need to examine sedentary behaviour in different contexts to fully understand how SEP influences behaviour. Further research should combine self-report and objective data to examine associations with specific sedentary behaviours in the contexts within which they occur, as well as total sedentary time.

## Competing interests

The authors declare that they have no competing interests.

## Authors’ contributions

AC and AP conceived and led the study. PG coordinated data collection. RP conducted the analyses and wrote the initial manuscript. All authors contributed to the interpretation of data and helped in writing the manuscript. All authors read and approved the final manuscript.
